# Tele-monitoring system for intensive care ventilators in isolation rooms

**DOI:** 10.1038/s41598-023-42229-4

**Published:** 2023-09-14

**Authors:** Su Hyeon Kim, Hyo-Chang Seo, Sanghoon Choi, Segyeong Joo

**Affiliations:** 1 Department of Biomedical Engineering, Asan Medical Institute of Convergence Science and Technology, Asan Medical Center, University of Ulsan College of Medicine, Seoul, Republic of Korea; 2 Digital Therapeutics Research Center, Smart Healthcare Research Institute, Samsung Medical Center, Seoul, South Korea

**Keywords:** Biomedical engineering, Engineering

## Abstract

The COVID-19 pandemic and discovery of new mutant strains have a devastating impact worldwide. Patients with severe COVID-19 require various equipment, such as ventilators, infusion pumps, and patient monitors, and a dedicated medical team to operate and monitor the equipment in isolated intensive care units (ICUs). Medical staff must wear personal protective equipment to reduce the risk of infection. This study proposes a tele-monitoring system for isolation ICUs to assist in the monitoring of COVID-19 patients. The tele-monitoring system consists of three parts: medical-device panel image processing, transmission, and tele-monitoring. This system can monitor the ventilator screen with obstacles, receive and store data, and provide real-time monitoring and data analysis. The proposed tele-monitoring system is compared with previous studies, and the image combination algorithm for reconstruction is evaluated using structural similarity index (SSIM) and peak signal-to-noise ratio (PSNR). The system achieves an SSIM score of 0.948 in the left side and a PSNR of 23.414 dB in the right side with no obstacles. It also reduces blind spots, with an SSIM score of 0.901 and a PSNR score of 18.13 dB. The proposed tele-monitoring system is compatible with both wired and wireless communication, making it accessible in various situations. It uses camera and performs live data monitoring, and the two monitoring systems complement each other. The system also includes a comprehensive database and an analysis tool, allowing medical staff to collect and analyze data on ventilator use, providing them a quick, at-a-glance view of the patient's condition. With the implementation of this system, patient outcomes may be improved and the burden on medical professionals may be reduced during the COVID-19 pandemic-like situations.

## Introduction

The COVID-19 pandemic has affected millions of people worldwide, with approximately 590 million confirmed cases to date^1^. Despite vaccination efforts and public health measures, the number of COVID-19 infections continues to rise, with new mutant strains being discovered^2, 3^. COVID-19 primarily affects the respiratory system, causing severe symptoms that often require oxygen support. Patients may experience complications, such as respiratory failure, acute respiratory distress syndrome, sepsis, and septic shock^4–7^. Ventilators are critical life support devices for severely ill COVID-19 patients, as their lungs are often damaged, leading to breathing difficulties^8^. For patients requiring intensive care, intensive care units (ICUs) equipped with mechanical ventilation and accessories, infusion pumps, and patient monitors are essential for improving their condition^9, 10^.

The use of multiple life support systems in the ICU is crucial for managing COVID-19 patients and improving their condition^11–13^. However, the operation of these devices in isolation ICUs requires medical staff to wear personal protective equipment (PPE) to protect themselves from the risk of infection^14–16^. Moreover, in addition to monitoring a patient, medical staff must check the devices in the isolation ICU to monitor key data that is not provided by the patient monitor.

Various image processing techniques have been used in many tasks for denoising, registration, and object detection^17^. Recently, in medical tele-monitoring systems, these techniques are adopted by combining a camera system for remote monitoring^18, 19^. The camera system enables the monitoring of patients’ condition and medical devices such as ventilator. Particularly, the ventilator panels need to be monitored in isolated ICUs.

Previous studies have proposed the use of tele-monitoring to avoid physical contact with patients and minimize the risk of staff infection. Vagvolgyi et al. developed a tele-monitoring system using a Maquet Servo-u ventilator (Getinge AB, Gothenburg, Sweden) linked to a touch screen and single camera, enabling real-time monitoring from outside the ICU^20^. Ng et al. monitored pressure and flow waveform data from a PB980 ventilator (Medtronic, Dublin, Ireland) and used numeric data obtained from the wave data^21^. Yang et al. employed a YuMi collaborative robot (ABB, Switzerland) to deliver internal ICU images to medical staff outside the ICU^22^. Szlavecz et al. developed CURE Soft that enabled the real-time calculation of model-based respiratory mechanics for mechanically ventilated patients. CURE Soft has following two modes of operation: (1) Online mode: real-time monitoring decision support; (2) Offline mode: for user education purposes, auditing, or reviewing patient care^23^. John et al. developed a tele-monitoring system using the RS232 serial communication port from a GE Datex S/5 series monitor (GE Datex Ohmeda, Helsinki, Finland); however, it lacked a database and analysis tool^24^. Battista proposed a novel remote monitoring system for a long-term home-based ventilation therapy, together with two configurations of the ventilation monitoring unit placed at the patient’s residence^25^. Seifert et al. developed a prototype of an interface tool for remote pediatric-critical-care ventilator. It allowed physicians and respiratory therapists to view real-time ventilator data^26^. Rehm et al. manufactured and monitored systems using mobile applications, whereas Seddik et al. developed systems using Arduino boards and IP cameras^27, 28^. As such, systems capable of remote monitoring from residence have also been developed.

In this study, we aimed to develop a multi-modal tele-monitoring system for ventilators in isolation ICUs. Our system used two cameras to monitor the ventilator screen and collected curve, breath, setting, and alarm channels data from the Maquet Servo-i ventilator (Getinge AB, Gothenburg, Sweden) in real time. In addition, our system included a database with access to patient data and analysis tools.

## Methods

**Figure 1:**
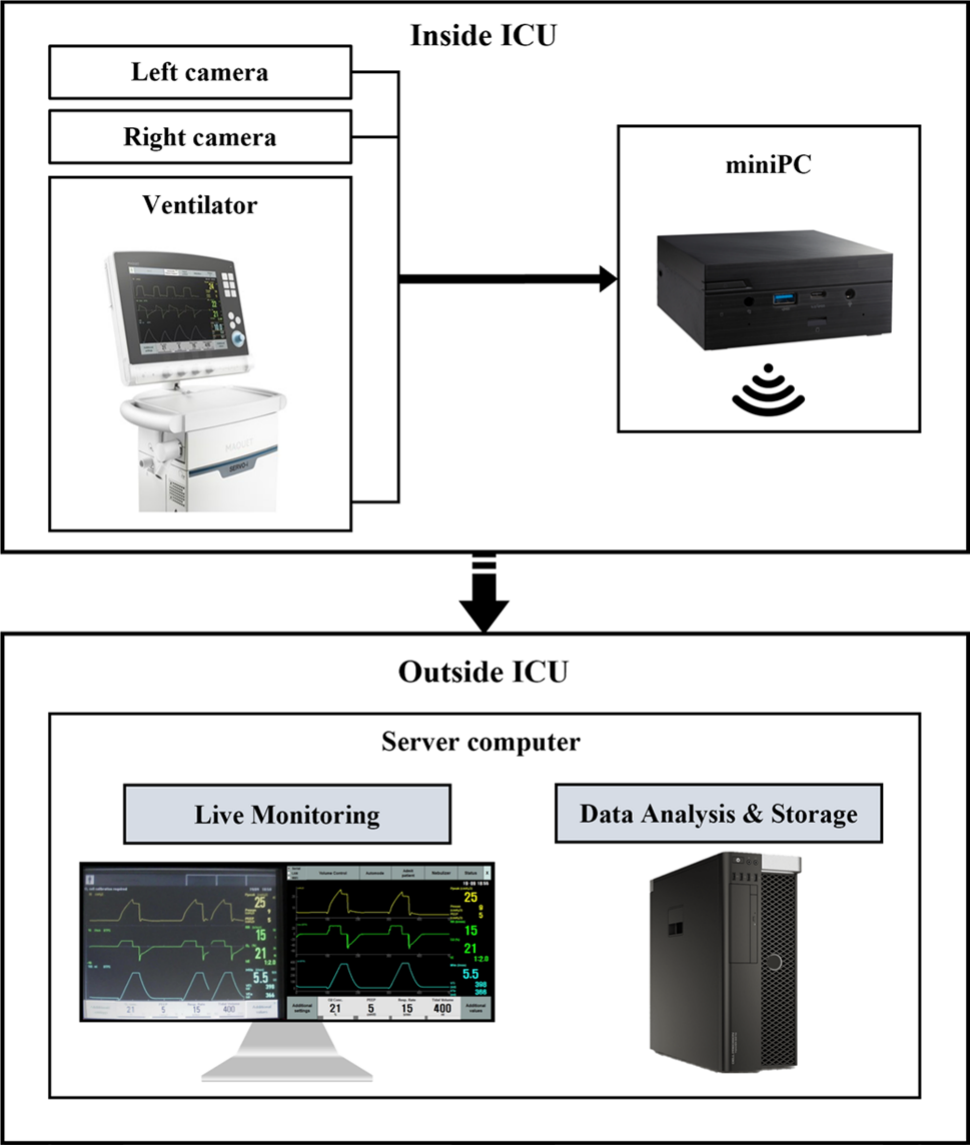
System overview.

The proposed multi-modal usable tele-monitoring system for the isolation ICU consisted of three parts: a medical device panel with video image processing facilities, a transmission part with a packet that included video data and patient respiratory data, and a tele-monitoring part comprising of a real-time viewer, a database, and analysis tools. The system was connected to the ventilator, one of the most commonly used equipment in the ICU (Fig. 1).

**Figure 2:**
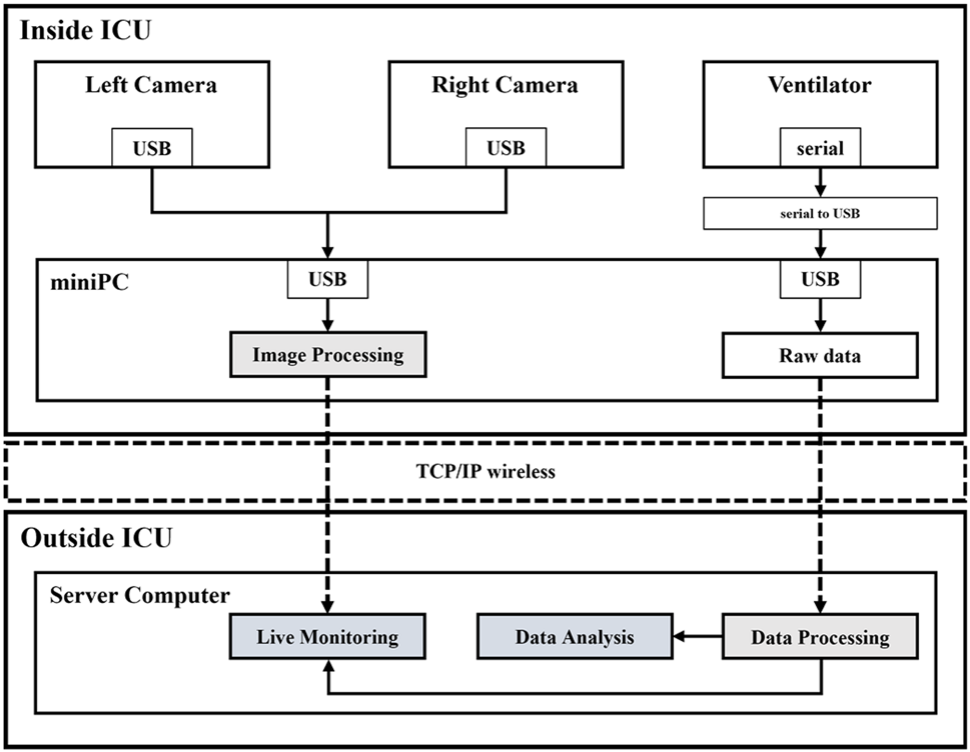
System architecture.

To enable remote access, a miniPC was set up for both LAN-based (wired) and WiFi-based (wireless) communication. Dual cameras were connected to the bottom of the ventilator monitor and directly to the miniPC via a USB port. The miniPC was connected to a server computer to establish the tele-monitoring system. Figure 2 provides an overview of the components of the tele-monitoring system, including the medical device panel with dual cameras for video image processing and the transmission part with a packet that includes video data and patient respiratory data. The tele-monitoring part comprises a real-time viewer, database, and analysis tools. Medical staff outside the ICU can remotely access the data received from the miniPC through the server computer. The specifications of the cameras used in the system are listed in Supplementary Table S2.

### Communication

**Figure 3:**
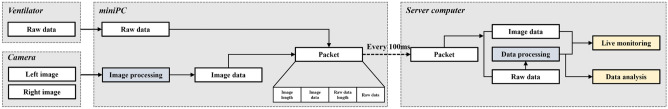
Communication structure.

The tele-monitoring system was developed based on the Python 3.9 programming language. For Servo-i ventilators, the external equipment can be connected via the RS232C serial interface. The ventilator supports baud rate 9600, data format ASCII or binary, and software handshake. The miniPC (PN51E1-B, ASUS, Taiwan) was manufactured by ASUS, and a CPU of AMD Ryzen 75700U with 32 GB RAM was employed. The miniPC and server computer were connected via Transmission Control Protocol/Internet Protocol (TCP/IP) communication. The miniPC transmits the image processed video of the ventilator screens captured by the dual cameras and raw data received from the ventilator to the server computer. Images were compressed by JPG to prevent data loss, and data of 800 × 600 images, such as the sizes of the image and ventilator screen, respectively, were transmitted using TCP/IP communication protocols. After image processing, image data were transmitted to the miniPC every 10 fps (100 ms), and data received from the ventilator during image processing were configured and transmitted in packets. Curve data were monitored by implementing the pressure, flow, and volume data as a real-time graph at a sampling rate of 50 Hz (20 ms). The packet was composed of image length, image data, raw data length, and raw data. Figure 3 shows a visual representation of the system.

### Ventilator data description

The tele-monitoring system communicated with the ventilator through a computer interface emulator (CIE) protocol provided by Maquet. The data from the ventilator were divided into four types: curve channel with several waveform, breath channel with numeric, setting channel with ventilator numeric, and alarm channel with numeric and character types that include the time of occurrence, description, and sound. Although 180 channels could be extracted from the ventilator, only 79 major channels were required and extracted owing to data traffic limitations. The extracted data are summarized in Table 1 and the important channels are shown in bold. Experienced staff from respiratory medicine participated in selecting the important ventilator data channels. This study was approved by the Institutional Review Board of the Seoul Asan Medical Center Hospital (IRB 2021-2864). Direct data on patients were not used in the study.

**Table 1:** Channel configurations.

Channel type	Channel name
Curve channel	**Airway pressure**
	**Volume**
Breath channel	**Measured breath frequency**
	**Exp. tidal volume**
	**Insp. tidal volume**
	**Insp. minute volume**
	**Exp. minute volume**
	**Peak pressure**
	**Mean airway pressure**
	**Pause pressure**
	**End exp. pressure**
	**O**_**2**_** concentration**
	**I:E ratio**
	**NIV, Leakage fraction**
	**Total PEEP**
Setting channel	**CMV frequency**
	**Pause time**
	**Insp. rise time**
	**Pressure control level above PEEP**
	**Pressure support level above PEEP**
	**PEEP**
	**Ventilation mode**
	**Insp./exp. pause hold, oxygen breaths/start breaths**
	**CPAP**
	**O**_**2**_** concentration**
	**I:E ratio**
	**Tidal volume**
	**Backup RR**
	**Insp. time in seconds**
	**Cycle off fraction level**
Alarm channel	**Exp. minute volume-alarm**
	**Apnea alarm/backup ventilation**
	**Gas supply alarm**
	**Battery alarm**
	**Power failure-alarm**
	**Mains failure-alarm**
	**No patient Effort**
	**Check tubing**
	**Breath frequency High**
	**Breath frequency Low**
	**PEEP low**
	**PEEP high**
	**CPAP high**
	**CPAP low**
	**Exp. minute volume too high-alarm**
	**Exp. minute volume too low-alarm**

*Significant channels to be statistically analyzed are written in bold, and the entire channel check is provided in Supplementary Table S1.

To enable real-time communication, packet data were transmitted via the CIE protocol every 100 ms. The ventilator provided volume and pressure curve data only in extended mode, and the flow data in the form of curved channels were calculated in real time from the received volume data. Each channel was stored as a CSV file every minute.

### Image processing

**Figure 4:**
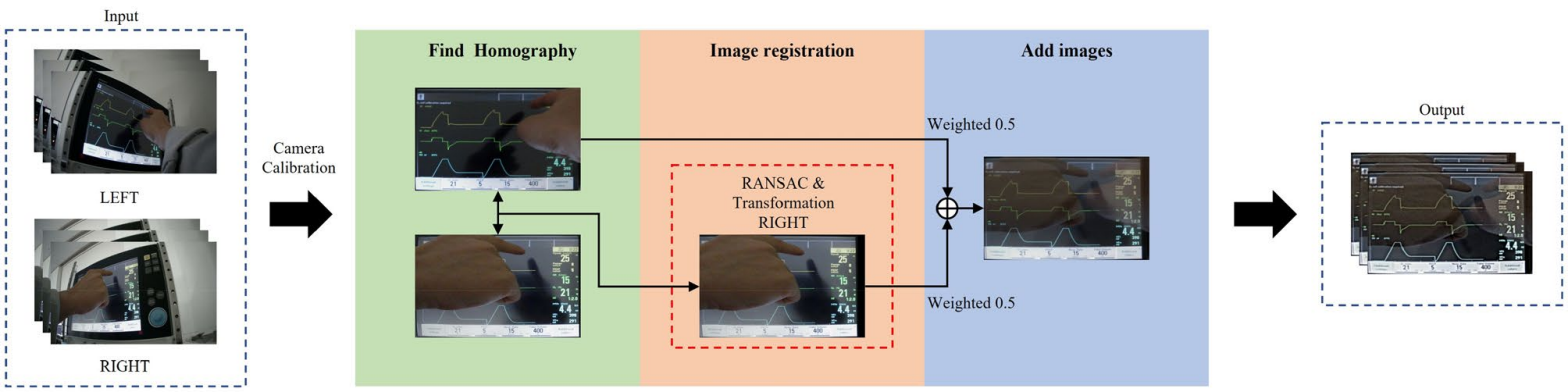
Flow chart of image processing.

The camera used in the system had a low light angle camera module (Arducam) and could capture video with a resolution of 1920 × 1080 at 30 fps. The camera is based on a 1/2.8" Sony IMX291 image sensor with a resolution of 2MP (1945H × 1109 V). Also, the camera had a field of view of 100° and focusing range of 3.3 ft to infinity. The video image processing involved four steps; each step was performed in 100 ms for real-time display, resulting in a flattened video image with minimal blind spots. As shown in Fig. 4, the processing steps include *Camera Calibration* and *Find homography* using inverse perspective transformation, *Image Registration*, and *Add Images*. The first step involves correcting the radial distortion caused by the camera lens using a check board captured from multiple perspectives to measure and correct the internal or external parameters through inverse calculation^29^. The *Camera Calibration* function provided by OpenCV was used for this purpose.

**Figure 5:**
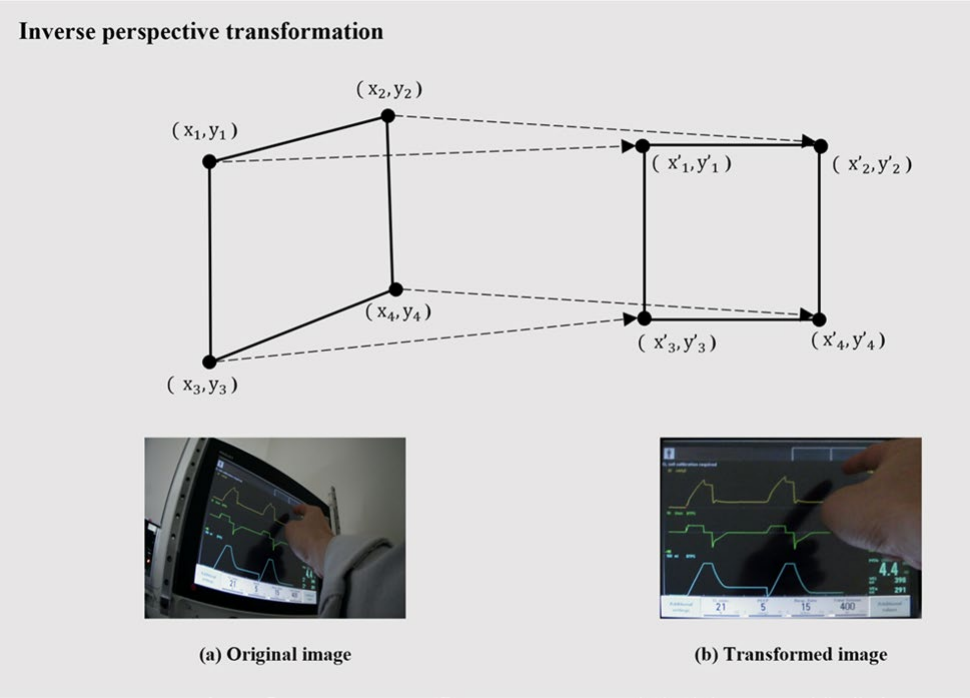
Inverse perspective transformation.

**Figure 6:**
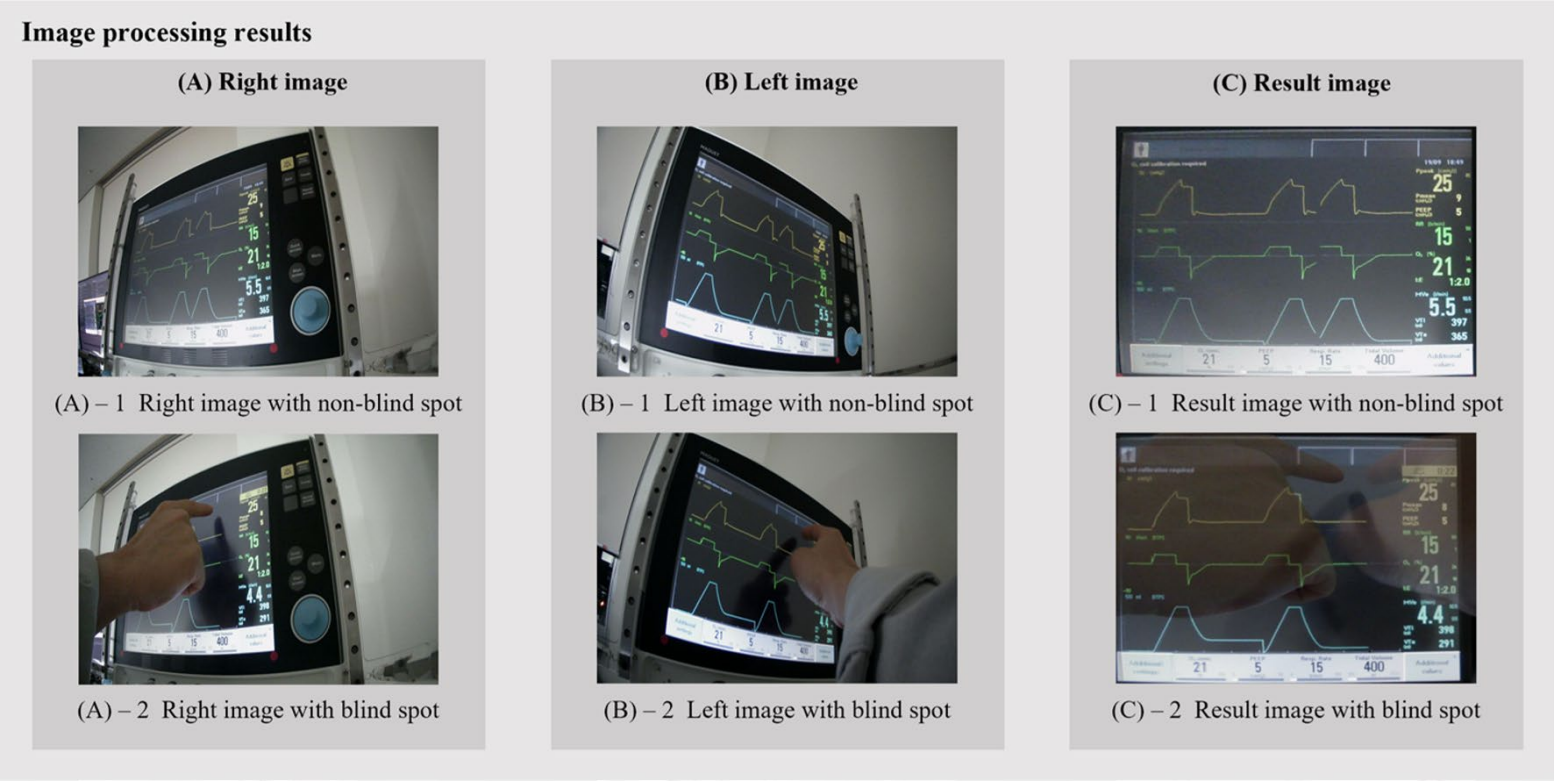
Image processing results.

In the second step, to transform the original image, an inverse perspective transformation was used. First, the four-corners of the ventilator panel in the original image (Fig. 5a) were identified and marked (Fig. 5)^30^. Using these corner positions, the transformed image (Fig. 5b) was generated through the application of inverse perspective transformation (as described in Eq. (1) below)^31^.

1 $$w\left[\begin{array}{c}{x}^{\mathrm{^{\prime}}}\\ {y}^{\mathrm{^{\prime}}}\\ 1\end{array}\right]=\left[\begin{array}{ccc}a& b& c\\ d& e& f\\ g& h& 1\end{array}\right]\left[\begin{array}{c}x\\ y\\ 1\end{array}\right],$$

where $(x,y)$ are the coordinates of the original image, and $({x}{\prime},y{\prime})$ are the moved coordinates based on the inverse perspective transformation. In the third step, we aimed to improve the precision of our image by aligning the left and right images through image registration. We employed the RANSAC (Random Sample Consensus) algorithm as our image registration method^32^. The algorithm analyzed the pixel values of both images and identified common features by applying the least squares method. The transformed matrix coefficients were then obtained based on these features, ensuring consistency between the positions of features at different points. In the final step, we combined the two images by multiplying each pixel value by a weight of 0.5. This resulted in the panel remaining the same; however, the differences or obstacles were obscured, thereby partially solving the issue of information loss. Figure 6 shows the results of image processing.

### Graphic user interface

We created two graphical user interfaces (GUIs) to monitor and analyze data in real time. We used PyQt, a python library that allowed the Qt framework to be used in python code for developing these interfaces.

**Figure 7:**
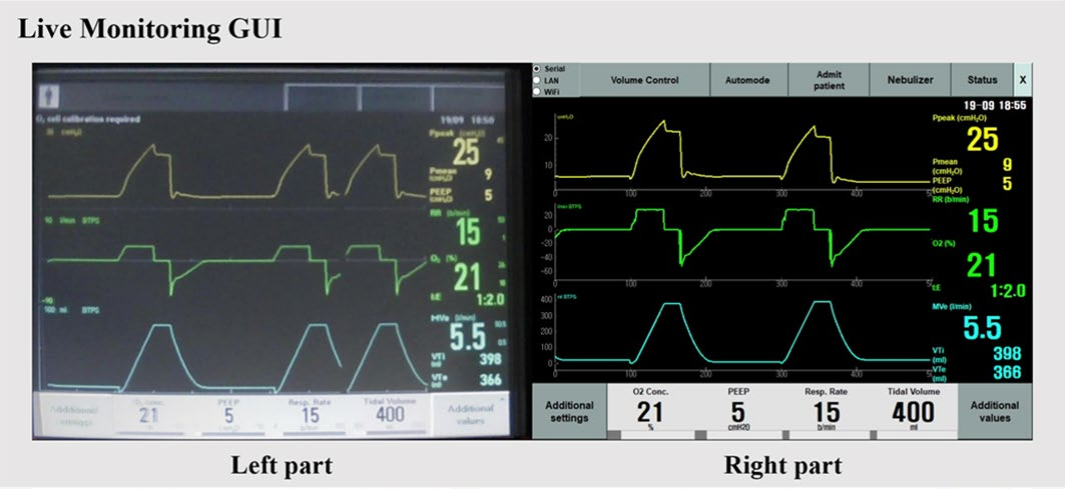
Live monitoring GUI. Numerical data, such as peak pressure, mean pressure, peep, respiratory rate, FiO_2_ concentration, inspiratory and expiratory ratio, expiratory minute volume, inspiratory tidal volume, expiratory tidal volume, and waveform data, can be monitored in real time.

Figure 7 shows the live monitoring GUI, which is divided into two parts. The left part shows images obtained using two cameras with minimized blind spots. Each image was processed to find homography and image registration and then combined appropriately. These procedures were performed by the miniPC and then sent to the server computer at a rate of 10 fps (100 ms). The right part in Fig. 7 shows the data received via communication with the Servo-i ventilator, which is displayed in the same format as the Servo-i ventilator screen. This makes it convenient to operate the ventilator without any difficulty.

**Figure 8:**
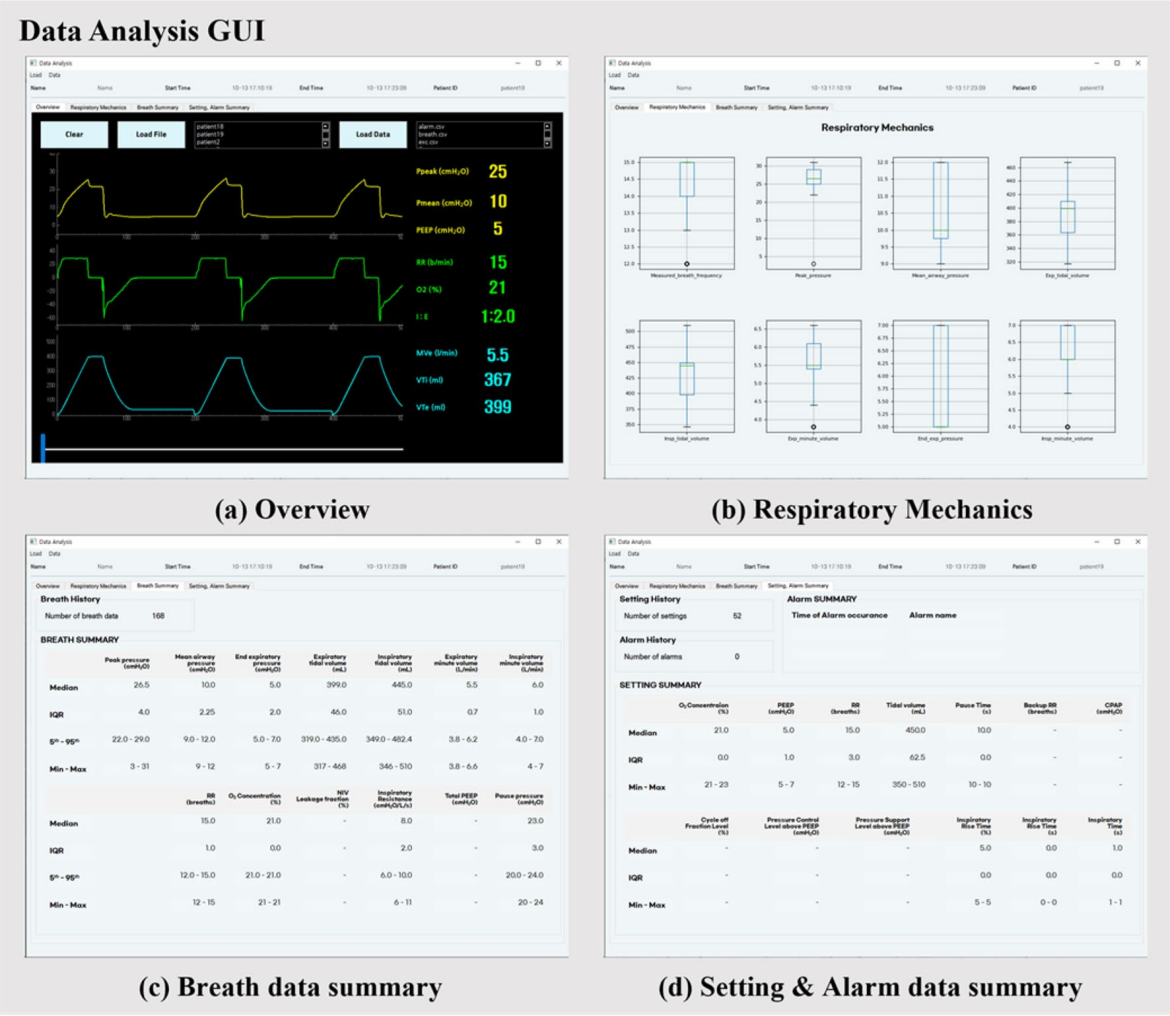
Data analysis GUI. (**a**) Overview tab allows users to view stored patient data and move the slider to view the entire data. It can be used to monitor patient information, start time, and end time to save ventilator data; (**b**) respiratory mechanics tab shows the respiratory mechanics via boxplots; (**c**,**d**) Breath data summary tab and Setting and Alarm data summary tab, respectively, allow statistical analysis, such as determination of median, interquartile range, minimum, and maximum of the main data of stored breath and setting channels. *Hyphen indicates that no parameters that vary depending on the ventilation mode exist.

Figure 8 shows the data analysis GUI, which allows the user to view the channels listed in bold in Table 1. The data stored in CSV format was used to check previous data. The main channels, including 26 out of 79, used for statistical analysis are listed in Table 1. To analyze the data for these channels, we required various tools, such as box plots, median, interquartile range, 90% confidence interval, minimum, and maximum. If an alarm was triggered, the user could check the time and information to determine the type of alarm.

## Results

We developed a multi-modal system for tele-monitoring isolation ICUs that can be accessed in real time via both wired and wireless options. Additionally, we constructed a database in CSV format for each channel every minute, with a data analysis tool available for accessing the stored data.

Table 2 provides a comparison of our proposed system with those developed in previous studies. Although Vagvolgyi et al. used a touchscreen type ventilator with the Maquet Servo-u ventilator (Getinge AB, Gothenburg, Sweden) product, it had no knobs, and only one camera was used for monitoring. The system developed by Ng et al. could check the pressure and flow rate curve data and monitor metadata calculated based on the model. The system developed by John et al. only collected physiological data from the anesthesia monitor, GE Datex S/5. Yang et al. developed a voice and video conferencing system for telemedicine using YuMi collaborative robots. Szlavecz et al. developed CURE Soft that enables real-time calculation of model-based respiratory mechanics. CURE Soft communicated with the PB840 ventilator (Medtronic, Dublin, Ireland) through the RS-232 serial port for data collection and live monitoring. Battista proposed a novel remote monitoring system for a home-based ventilation monitoring unit placed at the patient’s residence. The system could detect various clinical events. It was developed to collect respiratory data using a machine ventilation device and monitor it through a smartphone or web page. Seifert et al. developed a prototype of an interface tool for remote pediatric-critical-care. A Pulmonetic systems LTV 1200 ventilator (Avante Health Solutions, Concord, USA) was enabled with a wireless cellular interface to make its settings and performance data accessible in real time over a secured wireless Internet connection.

**Table 2:** Comparison with previous systems.

	Device	Camera status	Data collection status	Live monitoring system	Data analysis system
Proposed system	Maquet Servo-i ventilator	√	√	√	√
Vagvolgyi et al.	Maquet Servo-u ventilator	√	–	√	–
Ng et al.	PB980 ventilator	–	√	√	√
John et al.	GE Datex S/5 series monitor	–	√	–	√
Yang et al.	No device	√	–	√	–
Szlavecz et al.	PB840 ventilator	–	√	√	√
Battista	Fabrication machine ventilation system	–	√	√	–
Seifert et al.	Pulmonetic systems LTV 1200 ventilator	–	√	√	√

To evaluate image processing, structural similarity index measure (SSIM) and peak signal-to-noise ratio (PSNR) were employed^33^. The SSIM score is used to measure the similarity between two images based on luminance, contrast, and structure of images and is calculated using Eq. (2) as follows:

2 $$SSIM\left(x,y\right)=\frac{\left({2\mu }_{x }{\mu }_{y}+{C}_{1}\right)\left(2{\sigma }_{xy}+{C}_{2}\right)}{\left({{\mu }_{x }}^{2}+{{\mu }_{y }}^{2}+{C}_{1}\right)\left({\sigma }_{x}^{2}+{\sigma }_{y}^{2}+{C}_{2}\right)},$$

$${C}_{1}=\left({k}_{1}L\right), { C}_{2}=\left({k}_{2}L\right), L={2}^{bit\,per\,pixel}-1$$

where ${\mu }$ and ${\sigma }^{2}$ represent mean and variance of the pixel values of the two images, respectively. ${\sigma }_{xy}$ represents the covariance of the two images, whereas ${k}_{1}$ and ${k}_{2}$ have default values of 0.01 and 0.03 in Eq. (2), respectively. The SSIM score ranges from 0 to 1. The closer the value to 1, the higher the performance. The PSNR score calculation method is used to evaluate image quality loss information. The score is calculated using the mean square error of each pixel as follows:

3 $$PSNR=10\,{log}_{10}\left(\frac{{MAX}^{2}}{MSE}\right)$$

The MAX value represents the difference between the maximum and minimum pixel values in the original image; the MSE value measures the mean square error between pixels of the original and distorted images.

We evaluated the two images combination cases to reduce the blind spots caused by obstacles. Both algorithms exhibited similar performance in terms of image overlay. For the first case, we compared a reference image that combined both side images without any obstacles to each side of the two images to measure their similarity. In the second case, we compared the same reference image with a combined image and each side of the two images with obstacles.

Table 3 lists the scores obtained in two cases. The SSIM values of the combined images in Case 1, when compared with the left and right images, exhibit good quality scores of 0.948 and 0.932 respectively, as compared to the original image. In Case 2, the left and right images exhibit SSIM values of 0.841 and 0.837, representing a difference of 0.107 and 0.095, respectively, compared to those in Case 1. Furthermore, the combined image exhibits an SSIM score of 0.901, showing approximately 4.5% improvement compared with images with and without obstacles on the left and right.

**Table 3:** Results of image processing.

	SSIM score		PSNR (dB)			
	Left	Right	Left	Right		
Case 1	0.948	0.932	23.402	23.414		

	SSIM score			PSNR (dB)		
	Left	Right	Combined	Left	Right	Combined
Case 2	0.841	0.847	0.901	15.736	17.062	18.130

SSIM and PSNR represent reconstruction information loss in the image for the two cases.

Regarding PSNR, values of 15 dB or less indicate poor quality, approximately 25 dB indicates normal quality, and 32 dB or more is considered good quality. In Case 1, the values of PSNR for the combined images, when compared with those of left and right images, are 23.042 and 23.414 dB, respectively, which are almost similar, confirming normal quality. In Case 2, the left and right images covered by obstacles exhibit PSNR values of 15.736 and 17.062 dB, respectively, indicating poor quality. These values are approximately 32.4 and 27.1% lower than those for Case 1 without obstacles. In contrast, the minimum blind spot algorithm shows improved image quality owing to restoration, with a PSNR value of 18.13 dB.

## Discussion

Our study was aimed to develop a flexible system that leverages the benefits of previously developed tele-monitoring systems and can be used in various medical environments. Although the developed system was limited to ventilator monitoring, it was constructed as multi-modal and expandable. A single panel could be configured for cases with or without data communication, and a dual panel could be established if each system has limitations. We suggested the use of both wired and wireless communication systems in the medical environment^34^. We also developed a dual camera system-based real-time system, which used the new algorithm for minimum blind spots caused by obstacles such as the hands of medical staff. The reconstructed image could help medical staff capture the field of view obscured by obstacles, such as robot or medical staff hands.

However, the developed system had several limitations. First, we used a camera with an FHD 1920 × 1080 resolution to obtain high-resolution video images. However, owing to image processing, this resolution was reduced to 800 × 600, reducing the sharpness of the images. In the future, we recommend using a camera with a higher resolution or enhancing the image sharpness through image processing techniques. Second, the ventilator led to data loss due to structural problems of sampling rate limitation, which was presumed to be an internal problem rather than related to the communication environment. To address this problem, different correction algorithms must be applied, and an accurate data loss rate must be recorded. In conclusion, our developed system has promising features for tele-monitoring systems, and it should be further assessed to determine whether this problem occurs in other equipment or only while monitoring ventilators.

## Conclusion

We developed a tele-monitoring system that included a dual camera-based minimal blind spot panel and a real-time information collection panel. This system was adaptable to various medical settings using wired and wireless communication system. Moreover, the system allowed for various retrospective analyses and log checking via a database to monitor the patient condition. For a device without a communication output port, the camera system could be used to operate it. However, if the equipment included a communication system, it could be integrated into a single panel. This system has the potential to be used extensively for monitoring various medical equipment. In future studies, we plan to extend this system to monitor other critical medical devices, such as implantation pumps, patient monitors, and ventricular assist devices, to assess its scalability.

### Supplementary Information

Supplementary Information.

## Data Availability

Direct data on patients were not used in the study. The datasets used and/or analyzed during the current study are available from the corresponding author on reasonable request.
