# Supplementary material for: Tele-monitoring system for intensive care ventilators in isolation rooms
Source: Sci Rep. 2023 Sep 14;13:15207. doi: 10.1038/s41598-023-42229-4 (PMC10502084; doi:10.1038/s41598-023-42229-4)
Supplement: Supplementary file 1 — Supplementary Information. [file 41598_2023_42229_MOESM1_ESM.docx]

Supplementary

## Entire channels configuration

The channel data extracted by the Servo-i ventilator is as follows. In the case of significant channels, they are written in bold, and statistical analysis is conducted.

Supplementary Table 1

Entire channel configuration.

| Channel type | Channel name |
| --- | --- |
| **Curve channel** | **Airway pressure** |
|  | **Volume** |
| **Breath channel** | **Measured breath frequency** |
|  | **Exp. tidal volume** |
|  | **Insp. tidal volume** |
|  | **Insp. minute volume** |
|  | **Exp. minute volume** |
|  | **Peak pressure** |
|  | **Mean airway pressure** |
|  | **Pause pressure** |
|  | **End exp. pressure** |
|  | **O2 concentration** |
|  | Insp. Resistance |
|  | **I:E ratio** |
|  | Ti (Insufflation time) |
|  | **NIV, Leakage fraction** |
|  | Elastance |
|  | Ti/Ttot |
|  | **Total PEEP** |
|  | Spontaneous Breath frequency |
|  | Work of Breathing, Ventilator |
|  | Work of Breathing, Patient |
| **Setting channel** | **CMV Frequency** |
|  | **Pause Time** |
|  | SIMV Frequency |
|  | **Insp. Rise Time** |
|  | **Pressure Control Level above PEEP** |
|  | **Pressure Support Level above PEEP** |
|  | **PEEP** |
|  | **Ventilation Mode** |
|  | **Insp./Exp. Pause Hold, Oxygen Breaths/Start Breaths** |
|  | **CPAP** |
|  | Exp. minute vol. Upper alarm limit |
|  | Exp. minute vol. Lower alarm limit |
|  | Upper pressure limit |
|  | Alarm mute/pre-mute Status |
|  | **O2 concentration** |
|  | Trigger sensitivity level below PEEP |
|  | Trigger sensitivity level above PEEP |
|  | **I:E Ratio** |
|  | **Tidal volume** |
|  | **Backup RR** |
|  | Backup Ti in Seconds |
|  | **Insp. Time in seconds** |
|  | Insp. Rise Time, in seconds |
|  | SIMV Breath duration |
|  | Back-up Pressure Level Above PEEP |
|  | Insp. flow |
|  | **Cycle off Fraction Level** |
|  | Breath frequency Upper alarm limit |
|  | Breath frequency Lower alarm limit |
|  | PEEP Lower alarm limit |
|  | PEEP Upper alarm limit |
|  | Exp. minute vol. Lower alarm limit |
|  | Backup I:E Ratio |
| **Alarm channel** | O2 conc. too high-alarm |
|  | O2 conc. too low-alarm |
|  | Airway pressure alarm Upper pressure limit exceeded-alarm |
|  | **Exp. minute volume-alarm** |
|  | **Apnea alarm/Backup ventilation** |
|  | **Gas supply alarm** |
|  | **Battery alarm** |
|  | **Power Failure-alarm** |
|  | **Mains Failure-alarm** |
|  | Barometer error-alarm |
|  | High continuous pressure-alarm |
|  | Overrange-alarm |
|  | **No patient Effort** |
|  | Leakage out of range |
|  | **Check tubing** |
|  | **Breath frequency High** |
|  | **Breath frequency Low** |
|  | **PEEP Low** |
|  | **PEEP High** |
|  | **CPAP High** |
|  | **CPAP Low** |
|  | **Exp. Minute volume too high-alarm** |
|  | **Exp. Minute volume too low-alarm** |
|  | Leakage fraction too high |

* Channels with significant statistical analysis are written in bold.

## Ablation study results

We proceeded with the ablation study which is an additional experiment based on the number of cameras with different positions of obstacle. The position of the camera is left bottom (LB), left top (LT), right bottom (RB), and right top (RT). As shown in Table 1, a slightly better performance is obtained using four cameras. Considering the inconvenience caused by using four cameras and to minimize cost, we selected the position of ‘LB+RB,’ which yielded the best performance with two cameras.

Supplementary Table 2

Results of additional experiment

| **Position of the cameras** | **LT+LB+RB+RT** | SSIM | | 0.80 | | 0.83 | | **0.86** | | **0.84** | | 0.81 | | **0.828** | |
| --- | --- | --- | --- | --- | --- | --- | --- | --- | --- | --- | --- | --- | --- | --- | --- |
|  |  | PSNR | | 16.78 | | 16.38 | | 17.55 | | **17.86** | | **16.21** | | **16.956** | |
|  | **LB+RT+LT** | SSIM | | 0.83 | | 0.71 | | 0.81 | | 0.82 | | 0.73 | | 0.78 | |
|  |  | PSNR | | 17.68 | | **16.69** | | 15.69 | | 16.60 | | 13.45 | | 16.022 | |
|  | **LB+RB+LT** | SSIM | | 0.85 | | 0.75 | | **0.86** | | 0.76 | | 0.80 | | 0.804 | |
|  |  | PSNR | | 17.10 | | 15.98 | | **18.3** | | 15.53 | | 16.4 | | 16.662 | |
|  | **LB+RT+RB** | SSIM | | 0.77 | | **0.85** | | 0.79 | | 0.73 | | 0.75 | | 0.778 | |
|  |  | PSNR | | 14.71 | | 16.10 | | 12.13 | | 12.96 | | 13.01 | | 13.782 | |
|  | **LT+RT+RB** | SSIM | | 0.75 | | 0.75 | | 0.72 | | 0.71 | | **0.84** | | 0.754 | |
|  |  | PSNR | | 14.63 | | 14.32 | | 12.18 | | 11.96 | | 15.55 | | 13.728 | |
|  | **RT+LT** | SSIM | | 0.71 | | 0.70 | | 0.75 | | 0.82 | | 0.81 | | 0.758 | |
|  |  | PSNR | | 12.00 | | 12.66 | | 15.81 | | 15.35 | | 15.55 | | 14.274 | |
|  | **LT+RB** | SSIM | | 0.85 | | 0.78 | | 0.71 | | 0.73 | | 0.82 | | 0.778 | |
|  |  | PSNR | | 15.91 | | 14.78 | | 13.56 | | 14.96 | | 16.10 | | 15.062 | |
|  | **RT+LB** | SSIM | | 0.72 | | **0.85** | | 0.72 | | 0.81 | | 0.75 | | 0.770 | |
|  |  | PSNR | | 12.12 | | 16.10 | | 14.13 | | 15.90 | | 14.25 | | 14.5 | |
|  | **LB+RB** | SSIM | | **0.90** | | 0.84 | | 0.77 | | 0.76 | | 0.75 | | 0.804 | |
|  |  | PSNR | | **18.13** | | 15.96 | | 16.37 | | 15.01 | | 15.54 | | 16.202 | |
|  | **Position of the obstacles** | | Right Top Corner | | Left Top Corner | | Middle Part | | Right Bottom Corner | | Left Bottom Corner | | Average | |  |
